# n-3 polyunsaturated fatty acids supplementation enhances hippocampal functionality in aged mice

**DOI:** 10.3389/fnagi.2014.00220

**Published:** 2014-08-25

**Authors:** Debora Cutuli, Paola De Bartolo, Paola Caporali, Daniela Laricchiuta, Francesca Foti, Maurizio Ronci, Claudia Rossi, Cristina Neri, Gianfranco Spalletta, Carlo Caltagirone, Stefano Farioli-Vecchioli, Laura Petrosini

**Affiliations:** ^1^Department of Psychology, University Sapienza of RomeRome, Italy; ^2^Lab of Experimental and Behavioral Neurophysiology, Santa Lucia FoundationRome, Italy; ^3^Department of Experimental and Clinical Sciences, University “G. D'Annunzio”Chieti, Pescara, Italy; ^4^Division of Information Technology, Engineering and the Environment, Mawson Institute, University of South AustraliaMawson Lakes, SA, Australia; ^5^Lab of Proteomic and metabonomic, Santa Lucia FoundationRome, Italy; ^6^Department of Experimental Medicine and Surgery, University Tor Vergata of RomeRome, Italy; ^7^Lab of Clinical and Behavioral Neurology, Santa Lucia FoundationRome, Italy; ^8^Department of Neuroscience, University Tor Vergata of RomeRome, Italy; ^9^Institute of Cell Biology and Neurobiology, National Research Council, Santa Lucia FoundationRome, Italy

**Keywords:** aging, omega-3 fatty acids, cognitive decline, hippocampus, neuroprotection

## Abstract

As major components of neuronal membranes, omega-3 polyunsaturated acids (n-3 PUFA) exhibit a wide range of regulatory functions, modulating from synaptic plasticity to neuroinflammation, from oxidative stress to neuroprotection. Recent human and animal studies indicated the n-3 PUFA neuroprotective properties in aging, with a clear negative correlation between n-3 PUFA levels and hippocampal deficits. The present multidimensional study was aimed at associating cognition, hippocampal neurogenesis, volume, neurodegeneration and metabolic correlates to verify n-3 PUFA neuroprotective effects in aging. To this aim 19 month-old mice were given n-3 PUFA mixture, or olive oil or no dietary supplement for 8 weeks during which hippocampal-dependent mnesic functions were tested. At the end of behavioral testing morphological and metabolic correlates were analyzed. n-3 PUFA supplemented aged mice exhibited better object recognition memory, spatial and localizatory memory, and aversive response retention, without modifications in anxiety levels in comparison to controls. These improved hippocampal cognitive functions occurred in the context of an enhanced cellular plasticity and a reduced neurodegeneration. In fact, n-3 PUFA supplementation increased hippocampal neurogenesis and dendritic arborization of newborn neurons, volume, neuronal density and microglial cell number, while it decreased apoptosis, astrocytosis and lipofuscin accumulation in the hippocampus. The increased levels of some metabolic correlates (blood Acetyl-L-Carnitine and brain n-3 PUFA concentrations) found in n-3 PUFA supplemented mice also pointed toward an effective neuroprotection. On the basis of the present results n-3 PUFA supplementation appears to be a useful tool in health promotion and cognitive decline prevention during aging.

## Introduction

The rise of life expectancy has amplified the interest in the prevention and improvement of age-related brain dysfunctions. In fact, cognitive deficits are hallmarks not only of pathological aging, as occurring in Alzheimer's disease and vascular dementia, but also of non-pathological aging processes (Kobayashi et al., 2010). Age-related cognitive decline is due to a progressive impairment of the underlying brain cell processes due to neuroinflammation, oxidative stress, reduced synaptic plasticity and neurogenesis, thus leading to a consequent and irreversible neuronal loss of gray and white matter volume (Driscoll et al., 2006; Masliah et al., 2006; Brown, 2009). As recently advanced, some of these neurodegenerative processes may be influenced by a targeted diet (Denis et al., 2013; Maruszak et al., 2014). Nutritional research indicates that Western diets do not provide the aged brain with an optimal supply of omega-3 polyunsaturated fatty acids (n-3 PUFA) (Woo, 2011). In fact, aging is associated to reduced cerebral n-3 PUFA levels due to reduced absorption, reduced n-3 PUFA capacity to cross the blood-brain barrier, reduced capacity to convert shorter chained fatty acids to longer fatty acids, and increased oxidative stress (Yehuda, 2012).

Although epidemiological studies suggested that high n-3 PUFA dietary intake slows the age-related cognitive decline (Fotuhi et al., 2009; Solfrizzi et al., 2010; Karr et al., 2011; Denis et al., 2013), confounding factors, as socio-economical status or healthy habits, make it difficult to isolate the specific protective impact of n-3 PUFA-enriched diet on cognitive function (Denis et al., 2013). Actually, the n-3 PUFA action in preventing age-related cognitive decline has been usefully addressed by using animal models that offer better possibilities of controlling over confounding factors and dietary manipulations (Luchtman and Song, 2013). Namely, in rodents n-3 PUFA deficiency may be associated with memory deficits and hippocampal plasticity reduction, while n-3 PUFA supplementation may improve learning and memory abilities, and neurogenic and synaptogenic functions (Fedorova and Salem, 2006; Hooijmans et al., 2012; Denis et al., 2013; Luchtman and Song, 2013).

Within the sparse literature on the cognitive enhancement by n-3 PUFA in aging, no study has definitively detailed the associations among cognition, hippocampal neurogenesis and volumes, and metabolic correlates in the same non-pathological aged animals. Thus, we investigated whether n-3 PUFA supplementation in aged mice was able to counteract or at least mitigate age-related cognitive decline and neurodegenerative processes, to enhance hippocampal neurogenesis, volume and neuronal density, and to ameliorate metabolic functions and lipid membrane composition. To this aim aged mice were given n-3 PUFA mixture, or olive oil or no dietary supplement for 8 weeks during which learning and memory abilities were investigated by means of a battery of behavioral tests assessing different facets of hippocampal-dependent functions. n-3 PUFA supplementation effects on hippocampal neurogenesis, volume, neuronal density, glial reactivity, and lipofuscin accumulation were analyzed. Amino acids and carnitines levels in the peripheral blood, as well as brain lipid concentrations were also measured.

## Materials and methods

### Animals

**Figure 1:**
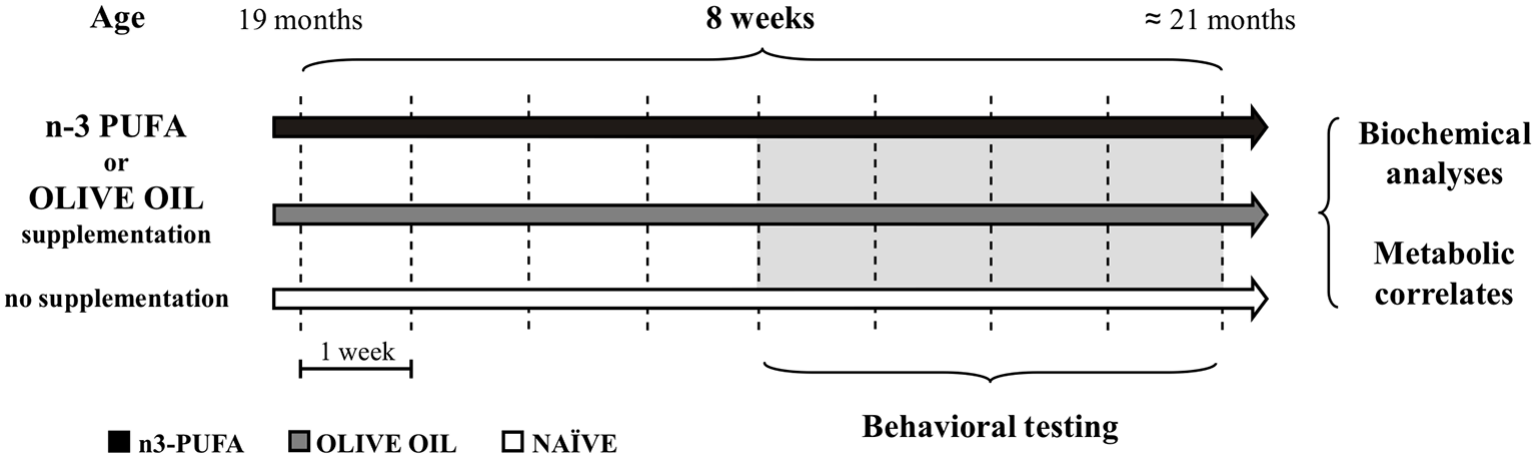
**Global timing of the experimental procedure**. Experimental groups of aged mice (n-3 PUFA, OLIVE OIL, and NAÏVE), type of dietary supplementation (duration: 8 weeks), behavioral testing (EPM, Elevated Plus Maze; SYM, Spatial Y-Maze; MWM, Morris Water Maze; ORT, Object Recognition Maze; CFC, Contextual Fear Conditioning), morphological analyses (hippocampal volumes, cell density, caspase-3 levels, astroglia and microglial cells, lipofuscin concentrations, neurogenesis) and metabolic correlates (blood amino acids and carnitines levels; brain n-3 PUFA levels) are indicated.

Male aged C57B6/J mice (19 month-old at the onset of study; 33.7 ± 0.3 g) were used in the present research (Charles River Laboratories, Italy). The animals were group-housed (three-four mice/cage) with temperature (22–23°C) and humidity controlled (60 ± 5%), under a 12:12 h light/dark cycle with free access to food and water. Animals were divided in three groups: (1) mice supplemented with an n-3 PUFA mixture (440 mg/kg) by daily gavage for 8 weeks (5 day/week) (Group name: n-3 PUFA; *n* = 12); (2) mice supplemented with olive oil (440 mg/kg) by daily gavage for the same period used as controls of an iso-caloric intake, as reported in previous studies (Kotani et al., 2006; Oarada et al., 2008; Nakamoto et al., 2010; Sinn et al., 2010; Danthiir et al., 2011; Vinot et al., 2011) (Group name: OLIVE OIL; *n* = 13); (3) no supplemented mice used as controls of eventual forced feeding effects (Group name: NAÏVE; *n* = 12) (Figure 1). Animals' weight was recorded weekly throughout the study. No significant differences among groups were found during the 8 treatment weeks [ANOVA (group × week): group: *F*_(2, 34)_ = 2.92, *p* = 0.07; week: *F*_(7, 238)_ = 2.53, *p* = 0.01; interaction: *F*_(14, 238)_ = 0.62, *p* = 0.85]. All efforts were made to minimize animal suffering and to reduce their number in accordance with the European Union Directive of September 22, 2010 (2010/63/EU). All procedures were approved by the Italian Ministry of Health.

### Food supplements

Food supplementation was performed by daily gavage to ensure that all cagemates received the same controlled amount of dietary supplements regardless of social hierarchy or appetitive drive.

n-3 PUFA group was supplemented with a mixture of fatty acids (Pfizer, Italy) containing high levels of n-3 PUFA, especially eicosapentaenoic acid (EPA), docosahexaenoic acid (DHA) and docosapentaenoic acid (DPA) (Table 1). OLIVE OIL group was supplemented with olive oil (Trasimeno, Italy) (Table 1). The three groups of animals were fed *ad libitum* with standard food pellets (Mucedola 4RF21 standard diet GLP complete feed for mice and rats; Mucedola, Italy) (Table 1).

**Table 1:** **Fatty acid composition of the dietary supplements (n-3 PUFA, olive oil) and standard diet**.

**Fatty acids**	**Percentage**
**n-3 PUFA MIXTURE**	
SFA	2.4
MUFA	8.0
PUFA	89.6
Of which n-3 PUFA:	**82.5**
EPA	51.8
DHA	21.2
DPA	3.3
SDA	2.4
ETA	1.4
HPA	1.2
ALA	1.2
**OLIVE OIL**	
SFA	15.0
MUFA	75.0
PUFA^*^	10.0
Of which n-3 PUFA:	**1.0**
ALA	1.0
**Nutrients**	**Percentage**
**STANDARD DIET**	
Cereals	66.5
Animal proteins	3.5
Vegetable proteins	18.2
Amino acids	0.1
Fodders	7.5
Vitamin and mineral mixtures	3.2
Fatty acids	0.4
Of which SFA	21.3
MUFA	22.0
PUFA^*^	56.7
Of which n-3 PUFA:	**8.6**
other nutrients	0.6

The fish-oil-based n-3 PUFA mixture contains mostly (≈83%) n-3 PUFA (EPA, eicosapentaenoic acid, 20:5 n-3; DHA, docosahexaenoic acid, 22:6 n-3; DPA, docosapentaenoic acid, 22:5 n-3; SDA, stearidonic acid, 18:4 n-3; ETA, eicosatetraenoic acid, 20:4 n-3; HPA, heneicosapentaenoic acid, 21:5; n-3 ALA, α-linolenic acid, 18:3 n-3) and low levels of MUFA. The n-3 PUFA mixture dose corresponds to ≈360 mg/kg of n-3 PUFA (Calviello et al., 1997). Conversely, olive oil contains mostly MUFA and has low levels of n-3 PUFA (1%).

* Indicates no presence of EPA, DHA and DPA. Abbreviations: SFA, saturated fatty acids; MUFA, monounsaturated fatty acids; PUFA, polyunsaturated fatty acids.

### Experimental procedures

After 4 weeks of dietary supplementation, mice were tested in the following validated behavioral tasks tapping distinct hippocampal-dependent abilities: Spatial Y-Maze (SYM) to assess spatial memory; Morris Water Maze (MWM) to evaluate short- and long-term localizatory memory; Object Recognition Test (ORT) to analyze novelty recognition memory; and lastly Contextual Fear Conditioning (CFC), to assess aversive associative learning. Furthermore, mice were tested in the Elevated Plus Maze (EPM) to measure anxiety levels.

After behavioral testing, a sample of 4 mice of each group received intraperitoneal (i.p.) BrdU injections daily for 6 days. Other 5 animals of each group were used to collect peripheral blood from the tail. At the end of these procedures, the animals were transcardially perfused and their brains were removed and cryopreserved at −80°C to perform immunocytochemistry and metabolic analyses.

### Behavioral testing

#### Elevated Plus Maze (EPM)

The maze was formed by a wooden structure in the shape of a cross with a central platform and four 35 × 6 cm arms raised 100 cm above the ground. The north and south arms were open, the east and west arms were enclosed by walls 20 cm high. During a 5-min trial the mouse was placed in the central platform and allowed to freely explore the maze. Since mice avoid open areas by confining movements to enclosed spaces or to the edges of a bounded space, a typical mouse tends to spend the majority of trial time in the closed arms. The entire apparatus was cleaned after each trial to remove olfactory cues. Trials were recorded by a ceiling-mounted camera and analyzed by a video analyzer (Ethovision XT, Noldus, The Netherlands). To evaluate anxiety level the following EPM parameters were measured: *total entries* and *total time* spent in the open and closed arms, *defecations* (Ruehle et al., 2013).

#### Spatial Y-Maze test (SYM)

The Y-maze apparatus made of gray Plexiglas consisted of three identical arms (8 × 30 × 15 cm) with a 120° angle between adjacent arms. The three arms were designated as: start arm (always open), in which the mouse started to explore the maze; familiar arm (always open); novel arm, blocked during the first trial and opened during the second trial (Ma et al., 2007). Several visual cues were placed on the inner walls of the maze. Y-maze test was performed in a dimly lighted room and consisted of two trials separated by a 1 h inter-trial interval (ITI). During the first trial (training phase) lasting 10 min the mouse was allowed to explore two arms (the start arm and familiar arm) with the novel arm being blocked. During the second trial (retention phase) lasting 5 min the three arms were opened and the mouse placed in the start arm was allowed to freely explore all three arms. Maze floor and walls were cleaned after each trial to remove olfactory cues. Trials were recorded by a ceiling-mounted camera and analyzed by a video analyzer (Ethovision XT, Noldus, The Netherlands). To evaluate the preference for the novel arm (novelty) the following parameters of retention phase were analyzed: *first entry* in the novel arm, *total entries, total distances* and *time* spent in the familiar vs. novel arm.

#### Morris Water Maze (MWM)

The mouse was placed in a circular white pool (diameter 140 cm) filled with 24°C water made opaque by the addition of atoxic acrylic white color (Giotto, Italy) (Carrié et al., 2002). An escape platform (diameter 5 cm) with a rough surface was placed in the middle of the NW quadrant 20 cm from the side walls. It was submerged 0.5 cm the water level. The pool located in a room uniformly lighted by four lamps (25 W each) was surrounded by several extra-maze cues. The water maze was surmounted by a video camera whose signal was relayed to a monitor and to the image analyzer (Ethovision XT, Noldus, The Netherlands). The protocol consisted of two phases. During the Place phase, mice were submitted to five consecutive sessions of three 60 s-trials, with 30 s-ITI for a total of 15 trials. Inter-session interval was 15 min during which mice were put in their home cages. At the beginning of each trial, mice were gently released into the water from pseudo-randomly varied starting points and were allowed to swim around to find the hidden platform. Mice that did not locate the platform within 60 s, were guided there by the experimenter. After the animals climbed to the platform, they were allowed to remain on it for 30 s. After 24 h, mice were submitted to the Probe phase, in which the platform was removed and the mice were allowed to search for it for 30 s.

To evaluate localizatory memory the following MWM parameters were analyzed: *latency* to reach the platform, mean *swimming velocity*, percentage of *time* spent in each of the four quadrants during Probe phase.

#### Object Recognition Task (ORT)

The apparatus consisted in a parallelepiped chamber made of transparent Plexiglas (56 × 42 × 21 cm). The task consisted of three trials, habituation, pre-test and test (De Bruin and Pouzet, 2006; Arsenault et al., 2011). During habituation, mice were introduced in the empty chamber and left to freely explore it for 10 min. Afterwards, they were put in their home cages for 5 min during which two identical objects (A1 and A2: 8 cm-diameter white Plexiglas disk vertically fixed to a white rectangular base) were placed in the chamber. During the pre-test, mice were allowed exploring the objects for 5 min. After a 1 h-ITI, animals were placed again for 5 min in the chamber, where one of the original objects was replaced by a novel one (B: 8 cm-diameter gray plastic sphere mounted on a squared transparent base). A video camera connected to a monitor and to the image analyzer (Ethovision XT, Noldus, The Netherlands) was placed on top of the apparatus. To evaluate the spontaneous tendency to contact the novel object (novelty) the following ORT parameters were analyzed: *contact time* with objects, *distance* traveled in the arena, mean *velocit*y, *rearings, grooming, defecation*s. A discrimination index was calculated: contact time with the novel object (T_no_) *minus* contact time with the familiar one (T_fo_)/total contact time with objects, $\frac{T_{\text{no}}-T_{\text{fo}}}{T_{\text{no}}+T_{\text{fo}}}$.

#### Contextual Fear Conditioning (CFC)

The apparatus consisted of a conditioning chamber (49 × 21 × 21 cm; Mod. 7532, Ugo Basile, Italy) with walls made of gray Plexiglas and ceiling made of transparent Plexiglas to allow video-recording. The grid-floor (steels spaced by 1.5 cm) was connected to a shock generator scrambler (conditioner 7531, Ugo Basile, Italy) (Cutuli et al., 2013).

CFC encompassed three sessions: baseline, training and context test. After a 3-min acclimation to the conditioning apparatus (baseline), the mouse received three foot-shocks (0.5 mA; 2 s) delivered with 60 s-ITI (training). After 24 h, it was placed again in the chamber for 6 min receiving no foot-shock (context test) (Crawley, 1999). Animals' behavior was recorded by a ceiling-mounted video camera and then analyzed through an image analyzer (Ethovision XT, Noldus, The Netherlands). To evaluate aversive learning the following CFC parameters were analyzed: *freezing duration* (considered as behavioral immobility, except for respiration movements) and *defecations*.

### Morphological analyses

#### BrdU treatment and immunocytochemistry

Four mice per group received daily i.p. injections of BrdU at a dose of 50 mg/kg BrdU dissolved in saline (0.9% NaCl adjusted to pH 7.2 with NaOH) for 6 days. One day after the final injection the animals were sacrificed and transcardially perfused, under deep anesthesia with chloral hydrate with 4% paraformaldehyde in 0.1 M phosphate buffer (PBS). The brains were removed and kept overnight in 4% paraformaldehyde (PFA). Afterwards, brains were equilibrated in 30% sucrose and cryopreserved at −80°C.

The hippocampus from brains embedded in Tissue-Tek OCT (Sakura, USA) was cut by cryostat at −25°C in 40 μm coronal serial free-floating sections. To perform BrdU detection, DNA was denaturated with 2N HCl for 40 min at 37°C to facilitate antibody access, followed by 0.1 M borate buffer pH 8.5 for 20 min. Sections were incubated overnight at 4°C with a primary antibody rat anti-BrdU (AbD Serotec Cat# MCA2060 RRID:AB_323427) diluted 1:300 in TBS containing 0.1% Triton, 0.1% Tween 20 and 3% normal donkey serum (blocking solution). For immunofluorescence analysis, sections were then stained for multiple labeling by using fluorescent methods. After permeabilization with 0.3% Triton X-100 in PBS, the sections were incubated with 3% normal donkey serum in PBS for 16–18 h with the following primary antibodies: 1:200 goat polyclonal antibodies against Doublecortin (DCX) (Santa Cruz Biotechnology, Inc. Cat# sc-8066 RRID:AB_2088494), 1:300 rabbit polyclonal antibodies against Glial Fibrillary Acidic Protein (Promega Cat# G5601 RRID:AB_430855), 1:150 rabbit monoclonal antibody against Ki67 (Lab Vision Cat# RM-9106-S RRID:AB_149707); 1:100 rabbit polyclonal antibody against Iba-1 (Wako Chemicals USA, Inc. Cat# 019-19741 RRID:AB_839504), 1:100 goat polyclonal antibody against Iba-1 (Abcam Cat# ab5076 RRID:AB_2224402); 1:100 rabbit polyclonal antibody against cleaved Caspase-3 (Cell Signaling Technology Cat# 9661S RRID:AB_331440). Secondary antibodies used to visualize the antigen were 1:200 donkey anti-rat Cy3-conjugated (Jackson ImmunoResearch; BrdU) 1:100, donkey anti-rabbit Cy2-conjugated (Jackson ImmunoResearch; Ki67, Iba-1), 1:100 donkey anti-goat Cy2-conjugated (Jackson ImmunoResearch Cat# 705-225-147 RRID:AB_2307341; DCX, Iba-1), 1:200 anti-rabbit Cy3-conjugated (Jackson ImmunoResearch; GFAP, Caspase-3).

Images of the immunostained sections were obtained by laser scanning confocal microscopy by using a TCS SP5 microscope (Leica Microsystem; Germany).

Analyses were performed in sequential scanning mode to rule out cross-bleeding between channels.

#### Quantification of cell numbers

Quantitative analysis of hippocampal cell populations was performed by means of design-based (assumption-free, unbiased) stereology. Slices were collected using systematic random sampling. The brain was coronally sliced in rostro-caudal direction, thus including the entire hippocampus. Approximately 40 coronal sections of 40 μm were obtained from each brain; about 1-in-6 series of sections (each slice thus spaced 240 μm apart from the next) were analyzed by confocal microscopy and (by unbiased stereological method) used to count the number of cells expressing the indicated markers throughout the rostro-caudal extent of the whole hippocampus. The total estimated number of cells positive for each of the indicated markers within the dentate gyrus (DG), CA1 and CA3 areas was obtained by multiplying the average number of positive cells per section by the total number of 40 μm sections comprising the entire DG, CA1 and CA3 (spaced 240 μm) (Jessberger et al., 2005; Kee et al., 2007; Farioli-Vecchioli et al., 2008).

Cells with lipofuscin deposits were recognized by their characteristic appearance and fluorescence of similar intensity in all three channels investigated (Kempermann et al., 2002). We counted only heavily loaded cells, in which the deposits obscured more than half of the nucleus.

An investigator blind to the experimental specimen performed cell count and proportional analyses. Cell number quantification was performed in at least 3 animals per group.

#### Volumetric measurements

Volumes of the DG, Ammon horn CA1 and CA3 and whole hippocampus were estimated by quantitative light microscopy using the Cavalieri's method (Pakkenberg and Gundersen, 1997). In brief, rostro-caudal sections from hippocampus of each animal (taking every sixth serial section) were mounted onto glass slide and stained with 4′,6-diamidino-2-phenylindole (DAPI) for 1 min. Stained sections were viewed at low magnification using Olympus BX53 digital photomicroscope. Digital images were then captured electronically and displayed on a computer screen. For each animal, DG, Ammon horn and whole hippocampus volumes were subsequently derived by multiplying the calculated mean surface area by the section thickness (40 μm) and the total actual number of sections in which the hippocampus was present.

#### Neuronal density measurements

Areal neuronal density values (number of neurons/mm^2^) were calculated within each of the hippocampal subfields (DG, CA3, and CA1) in the same tissue sections (stained with DAPI) that underwent the quantitative image analysis described elsewhere (Tandrup, 2004). Cells were counted within a rectangular area on a computer monitor, ranging in size from 1989 μm^2^ (60 × 35.15 μm) to 2270 μm^2^ (58.4 × 38.9 μm), and the rectangular area was superimposed onto the pyramidal cells of CA1 and CA3 areas and onto the granule cells of DG. The results were expressed as the cell density per mm^2^.

#### Dendritic growth of DCX^+^ neurons

Dendritic analysis of DCX^+^ neurons was performed by acquiring z-series of 15–25 optical sections at 1–1.5 lm of interval with a 40X oil lens, with the confocal system TCS SP5 (Leica Microsystem). Two-dimensional projections at maximum intensity of each z-series were generated with the LAS AF software platform (Leica Microsystem) in the TIFF format, and files were imported in the I.A.S. software (Delta Systems) to measure dendritic length. The number of branching points was counted manually in the same images. For each data point, 20–30 cells from 3 mice were analyzed (Farioli-Vecchioli et al., 2008).

### Metabolic correlates

#### Analysis of amino acids and carnitines by direct infusion mass spectrometry (DIMS)

Whole blood taken from the tail was collected on filter paper as dried blood spot (DBS), which is particularly suitable for small volume samples. The determination of amino acids (proline, valine, leucine/isoleucine/hydroxyproline, ornithine, methionine, phenylalanine, arginine, citrulline, tyrosine, glycine, alanine, serine, threonine, asparagine, aspartate, lysine/glicine, glutamate, histidine) and carnitines (free carnitine, acetylcarnitine, propionylcarnitine, butyrylcarnitine, isovalerylcarnitine, 3-hydroxybutyrylcarnitine/malonylcarnitine, 3-hydroxyisovalerylcarnitine/methylmalonylcarnitine, glutarylcarnitine/3-hydroxyhexanoylcarnitine, adipylcarnitine, tetra-decenoylcarnitine, myristoylcarnitine, hexadecenoylcarnitine, palmitoylcarnitine, linoleylcarnitine, oleylcarnitine, stearoylcarnitine) was performed on filter paper card DBS samples by direct infusion mass spectrometry (DIMS) following a standardized high-throughput screening method as described elsewhere (Chace et al., 2002; Rossi et al., 2011). The analysis was performed in DBS samples by adding isotopically labeled internal standards, according to the principle of isotope dilution internal standardization. Briefly, filter paper disks containing approximately 3–3.2 μL of whole blood, were punched out from DBS samples and from the quality controls (QCs) using an automatic puncher, into a polypropylene microliter plate. 100 μL of the extraction solution containing the internal standards were added to each paper disk, and the plate was shaken in a thermo mixer (700 rpm, 45°C, 50 min). The internal standards as well as the extraction solution and the QCs were from the NeoBase Non-derivatized MSMS Kit (Perkin Elmer Life and Analytical Sciences, Finland) (Ostrup and Wallac, 1994; Food and Drug Administration, 2004). 75 μL from the content of each well were transferred to a new microliter plate. The plate was placed in the autosampler for analysis. Low and high blood spot QCs were run in replicate in each plate, before and after the real samples. The DIMS analysis for the evaluation of metabolite profile in DBS samples was performed using a Liquid Chromatography-tandem Mass Spectrometry (LC/MS/MS) system consisting of an Alliance HT 2795 HPLC Separation Module coupled to a Quattro Ultima Pt ESI tandem quadrupole mass spectrometer (Waters Corporation, USA) (Sirolli et al., 2012; Bonomini et al., 2013; Rizza et al., 2014). The instrument was operated in positive electrospray ionization mode, using MassLynx V4.0 Software (Waters) with auto data processing by NeoLynx (Waters Corporation, USA). The 30 μL injections were made directly into the ion source through a narrow peek tube with a total run time (injection-to-injection) of 1.8 min. The mass spectrometer ionization source settings were optimized for maximum ion yields for every analyte. The capillary voltage was set to 3.25 kV, the source and desolvation temperature were 120°C and 350°C respectively, and the collision cell gas pressure was 3–3.5 e-3 mbar Argon (Sirolli et al., 2012; Bonomini et al., 2013; Rizza et al., 2014).

#### Analysis of fatty acids by GC/MS

Fatty acids were extracted using the method reported by Folch (Folch et al., 1957) with slight modifications. Briefly, brains were homogenized in CHCl_3_/MeOH (2:1 v/v) to a final dilution of 20-fold of the original sample volume, assuming that the tissue has the same specific gravity of water. Heptadecanoic acid was used as internal standard. The resulting organic phase was evaporated to dryness in a speed-vac at room temperature and then derivatized with BSTFA-TMCS 99:1 v/v (Sigma-Aldrich, Italy) for 1 h at 60°C. Derivatized samples were transferred in the injection vial and added with 20% v/v of Acetone. GC/MS analyses were performed using a Focus GC (Thermo Scientific, USA) equipped with 30 m × 0.25 mm fused silica capillary column SLB™-5MS (Supelco) and connected to a PolarisQ mass spectrometer (Thermo Scientific, USA). 2 μL of samples were injected in split mode (1:10 ratio), the injector temperature was set at 200°C; the carrier gas was Helium and the flow rate was maintained constant at 1 ml/min. The initial oven temperature of 100°C was held for 1 min and then raised to 250° C at 10°C/min and maintained for 6 min. After then the oven temperature was increased up to 310°C at 20°C/min and held for 5 min. Mass transfer line was maintained at 280°C and the ion source at 200°C. Analyses were performed in Selected Ion Monitoring (SIM) mode and fatty acids were identified by comparison with commercial standards.

#### Statistical analyses

All data were tested for normality (Shapiro-Wilk's test) and homoscedasticity (Levene's test) and presented as mean ± s.e.m. Behavioral data and metabolic correlates were analyzed by using one- and Two-Way ANOVAs (with group as between-factor and arm/session/quadrant as within-factor) followed by Newman-Keuls's tests. When parametric assumptions were not fully met, non-parametric analyses of variance (χ^2^ and Mann-Whitney *U*-tests) were used. Morphological data were analyzed by using Student's *T*-test. Values of *p* < 0.05 were considered significant (Statistica 7, Statsoft).

## Results

### Behavioral testing

#### Elevated Plus Maze (EPM)

As indicated by One-Way ANOVAs on entry frequencies [*F*_(2, 34)_ = 0.41, *p* = 0.67], time spent in the close vs. open arms [*F*_(2, 34)_ = 3.08, *p* = 0.06] and defecations [*F*_(2, 34)_ = 1.02, *p* = 0.37], the anxiety levels resulted similar for the three experimental groups, regardless of the food supplementation type.

#### Spatial Y-Maze (SYM)

**Figure 2:**
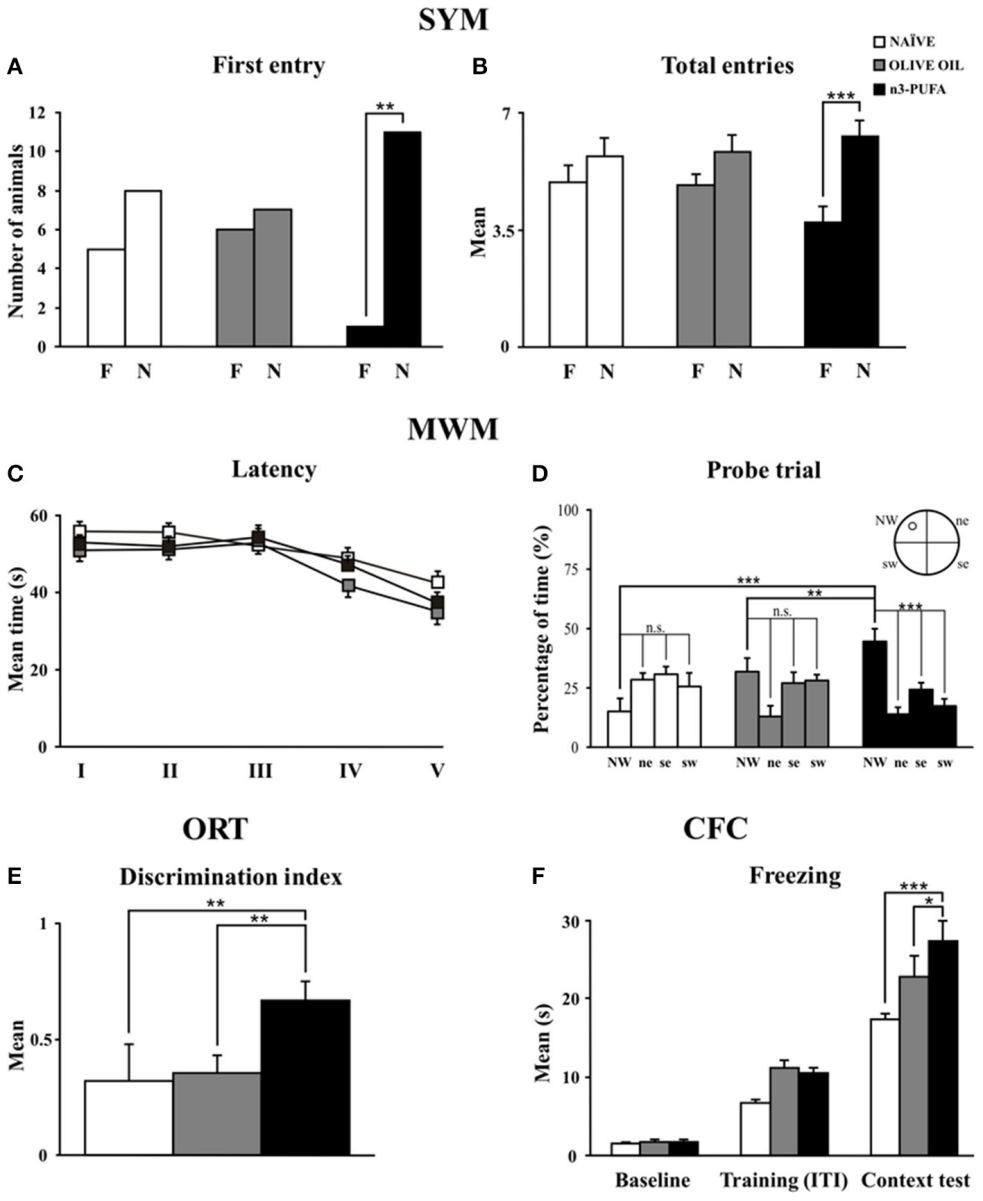
**n-3 PUFA effects on behavioral performances. (A,B)** First entry **(A)** and total entries **(B)** in the familiar **(F)** and novel (N) arm displayed by the three experimental groups in Spatial Y-Maze (SYM). **(C,D)** Mean escape latency to reach the hidden platform during Place phase of Morris Water Maze (MWM) **(C)** and time spent in the rewarded quadrant during Probe trial **(D)**. **(E)** Discrimination index in Object Recognition Test (ORT). **(F)** Mean freezing time exhibited during Baseline, Training (Inter-Trial Intervals: ITI) and Context Test of Contextual Fear Conditioning (CFC). Asterisks inside the graphs indicate the significance of comparisons between groups: ^*^p < 0.05, ^**^p ≤ 0.01, or ^***^p ≤ 0.001.

When allowed to choose between the novel and familiar arm, n-3 PUFA mice showed better discrimination abilities of spatial novelty as indicated by the higher number of animals first entering the novel arm and by the total entries in the novel arm. No motivational differences were observed among groups, given their similar total entries. Namely, analyses on frequency of first entry in the novel arm revealed that only n-3 PUFA mice showed a significant preference for it (χ^2^_1_ = 8.33, *p* = 0.004), while OLIVE OIL (χ^2^_1_ = 0.07, *p* = 0.78) and naïve (χ^2^_1_ = 0.69, *p* = 0.41) mice chose an arm by chance (Figure 2A). A Two-Way ANOVA (group x arm) on total entries revealed significant arm effect [*F*_(1, 35)_ = 32.05, *p* < 0.0001] and interaction [*F*_(2, 35)_ = 4.82, *p* = 0.01], while group effect was not significant [*F*_(2, 35)_ = 0.14, *p* = 0.87]. *Post-hoc* comparisons on interaction demonstrated that only n-3 PUFA group entered more the novel arm (*p* = 0.0001), while OLIVE OIL (*p* = 0.13) and NAÏVE (*p* = 0.09) groups equally entered the novel and the familiar arm (Figure 2B). No significant differences among groups were found on time spent in each arm [group: *F*_(2, 35)_ = 2.79, *p* = 0.07; arm: *F*_(1, 35)_ = 0.44, *p* = 0.51; interaction: *F*_(2, 35)_ = 2.56, *p* = 0.09] and total distances [group: *F*_(2, 35)_ = 1.04, *p* = 0.36; arm: *F*_(1, 35)_ = 11.07, *p* = 0.002; interaction: *F*_(2, 35)_ = 2.43, *p* = 0.10].

#### Morris Water Maze (MWM)

Regardless of the experimental conditions, all mice learned to localize the hidden platform as the Place sessions went by, as revealed by a Two-Way ANOVA (group × session) on latency values [group: *F*_(2, 34)_ = 2.68, *p* = 0.08; session: *F*_(4, 136)_ = 10.76, *p* < 0.0001; interaction: *F*_(8, 136)_ = 0.94, *p* = 0.49] (Figure 2C). Furthermore, no differences in mean swimming velocity among groups were found [*F*_(2, 34)_ = 0.28, *p* = 0.76]. A Two-Way ANOVA (group × quadrant) performed on time spent in the four quadrants during the Probe trial (24 h after Place phase) revealed significant quadrant effect [*F*_(3, 102)_ = 11.05, *p* < 0.0001] and interaction [*F*_(6, 102)_ = 7.07, *p* < 0.0001], while group effect [*F*_(2, 34)_ = 0.26, *p* = 0.77] was not significant. Interestingly, *post-hoc* comparisons revealed that only n-3 PUFA group swam significantly longer in the quadrant previously rewarded by the presence of the platform (n-3 PUFA vs. OLIVE OIL: *p* = 0.01; n-3 PUFA vs. NAÏVE: *p* = 0.001; OLIVE OIL vs. NAÏVE: *p* = 0.15) (Figure 2D). Time spent in the previously rewarded quadrant was significantly higher than the time spent in the other quadrants only in n-3 PUFA group (always *p* < 0.001).

#### Object Recognition Task (ORT)

**Figure 3:**
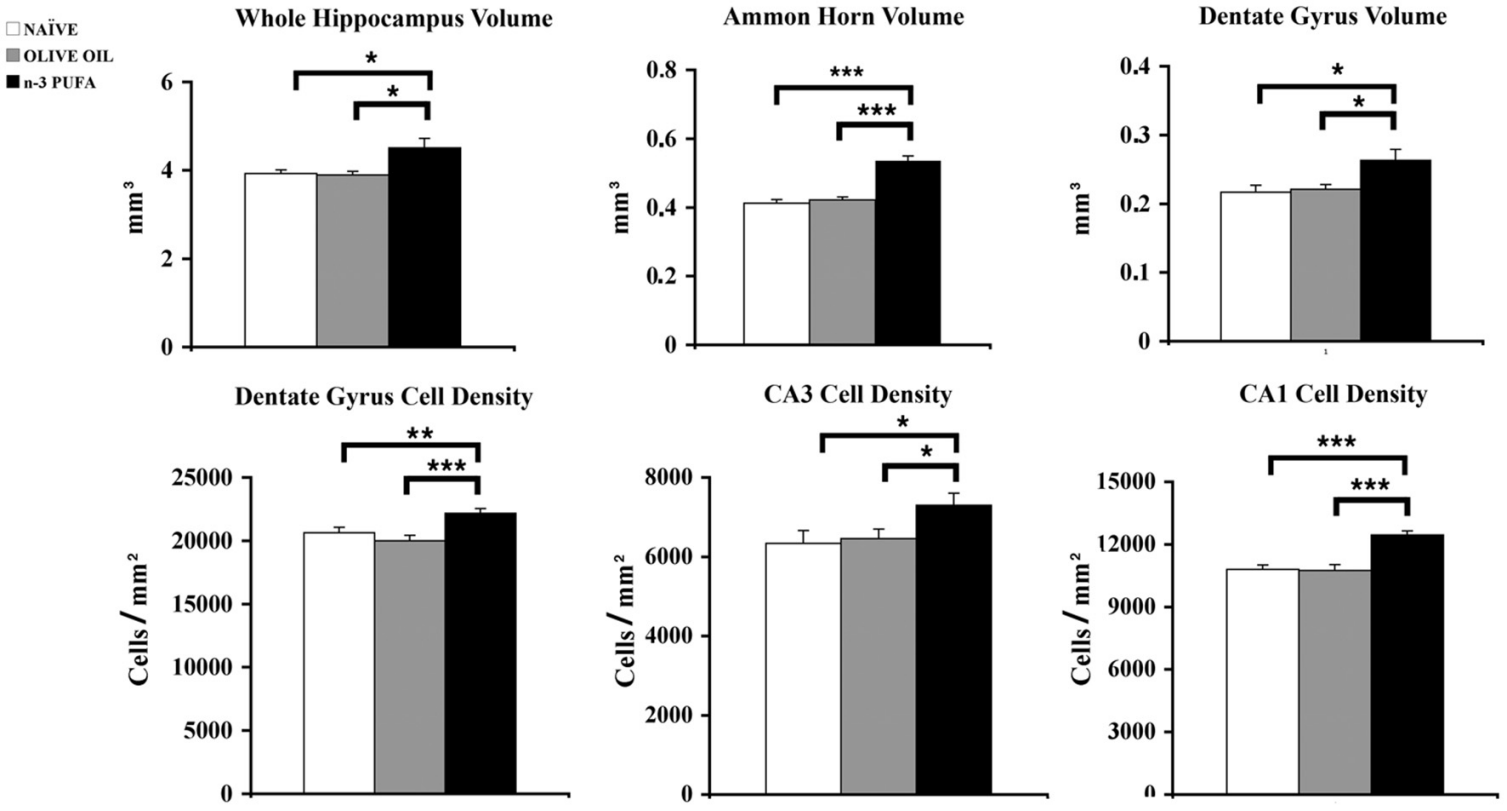
**n-3 PUFA effects on hippocampal volumes and cell density**. Volumes of whole hippocampus, Ammon Horn (CA1+CA3) and Dentate Gyrus resulted significantly enhanced in n-3 PUFA group in comparison to NAÏVE and OLIVE OIL groups. Also cell density was significantly increased in Dentate Gyrus, CA3 and CA1 of n-3 PUFA group in comparison to both control groups. ^*^p < 0.05, ^**^p < 0.01, or ^***^p < 0.001; Student's *T* test.

As indicated by the discrimination index (Figure 2E), n-3 PUFA group better recognized object novelty in comparison to both NAÏVE (Mann-Whitney U, *Z* = −2.40, *p* = 0.01) and OLIVE OIL (Mann-Whitney U, *Z* = −2.81, *p* = 0.004) groups. No significant differences among groups were found in emotional (grooming and defecations) as well as in explorative (distance, velocity and rearing) indices.

#### Contextual Fear Conditioning (CFC)

n-3 PUFA supplementation enhanced associative memory between aversive stimulus and environmental context, as indicated by the higher freezing behavior of n-3 PUFA group, a readout of increased memory. Namely, a Two-Way ANOVA (group × session) on freezing duration revealed significant group [*F*_(2, 34)_ = 6.93, *p* = 0.003] and session [*F*_(2, 68)_ = 191.07, *p* < 0.0001] effects. Also the interaction [*F*_(2, 68)_ = 3.39, *p* = 0.01] was significant. *Post-hoc* comparisons on interaction revealed that while during baseline and training all animals exhibited similar freezing levels, during context test n-3 PUFA group exhibited significantly higher freezing levels in comparison to OLIVE OIL (*p* = 0.02) and NAÏVE (*p* = 0.0001) groups (Figure 2F). Defecations did not differ among groups throughout the test [*F*_(2, 34)_ = 0.69, *p* = 0.51].

### Morphological analyses

#### Hippocampal volume and neuronal density

n-3 PUFA mice showed a total hippocampal volume significantly increased in comparison to OLIVE OIL (*p* = 0.03) and NAÏVE (*p* = 0.04) groups (Figure 3). To thoroughly analyze n-3 PUFA effect on hippocampal subfields, the volumes of Ammon Horn (CA = CA1 + CA3) and dentate gyrus (DG) were measured. In n-3 PUFA group CA volume significantly increased in comparison to OLIVE OIL (*p* = 0.0004) and NAÏVE (*p* = 0.0004) groups. Even in DG n-3 PUFA group exhibited enhanced volume in comparison to OLIVE OIL (*p* = 0.04) and NAÏVE (*p* = 0.04) groups.

To evaluate whether the volumetric differences were related to changes in neuronal density DAPI-stained nuclei were counted in DG, CA3, and CA1 hippocampal subfields (Figure 3). In DG n-3 PUFA supplementation resulted in a significant increase of granule cell density in comparison to OLIVE OIL (*p* = 0.005) and NAÏVE (*p* = 0.0001) groups. Even in CA3 pyramidal neuron density significantly increased in n-3 PUFA group in comparison to OLIVE OIL (*p* = 0.03) and NAÏVE (*p* = 0.03) groups. Finally, in CA1 n-3 PUFA group showed a significant increment in the pyramidal neuron density in comparison to OLIVE OIL (*p* = 0.0007) and NAÏVE (*p* = 0.0008) groups. All together, these data indicate that the n-3 PUFA-induced increase of hippocampal volumes was due to a decreased neuronal loss, as revealed by the increased neuronal density observed in the n-3 PUFA-treated aged mice.

#### Hippocampal apoptotic cell death

**Figure 4:**
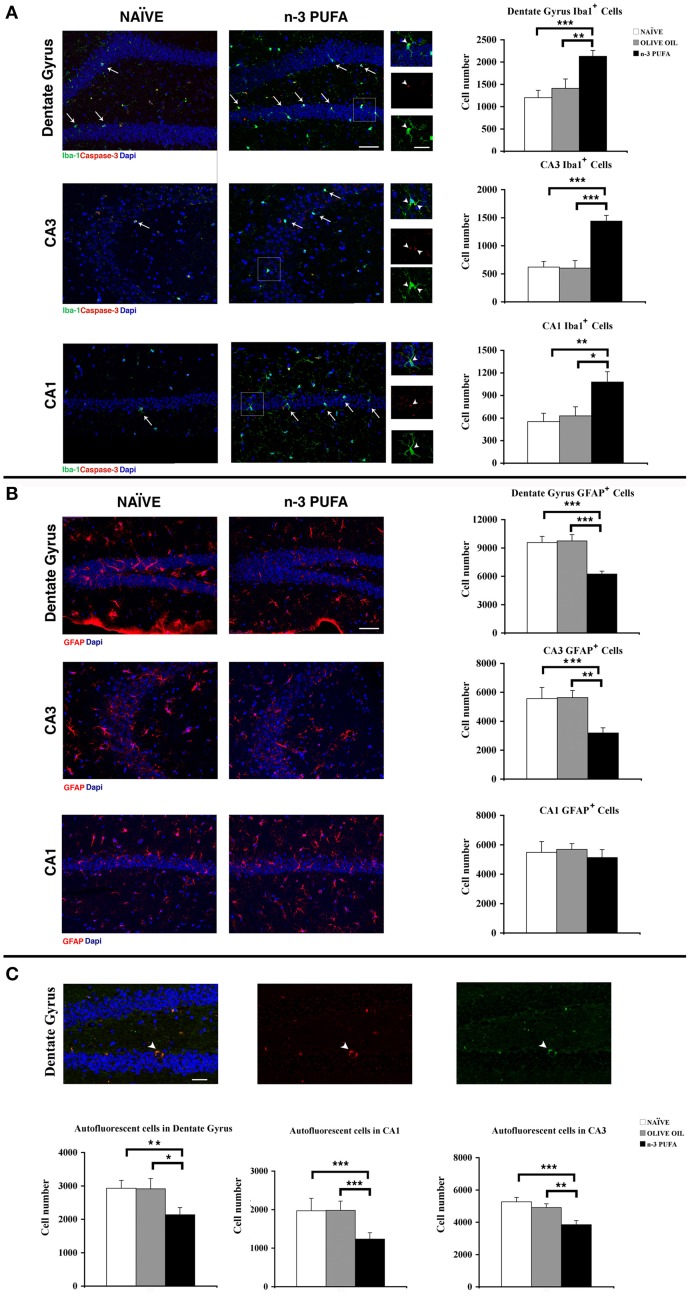
**n-3 PUFA effects on hippocampal glial populations and lipofuscin deposits. (A)** Representative fluorescence images of confocal optical sections of Dentate Gyrus, CA3 and CA1 in NAÏVE and n-3 PUFA groups, showing Iba-1^+^ (green, arrows) and caspase-3^+^ (red) cells. Scale bar 50 μm. In the central small panels, the higher magnifications (square boxes) show the neuronal caspase-3^+^ debris (red, arrowheads) phagocytated by the microglial cells (green, arrowheads). Scale bar 25 μm. The histograms on the right show the Iba1^+^ cell number of the Dentate Gyrus, CA3 and CA1 in n-3 PUFA, NAÏVE and OLIVE OIL groups. **(B)** Representative fluorescence images of confocal optical sections representing GFAP^+^ cells (red) in Dentate Gyrus, CA3, and CA1 of NAÏVE and n-3 PUFA groups, showing decreased astrocytosis in the n-3 PUFA group in Dentate gyrus and CA3 but not in CA1. Scale bar 50 μm. The histograms on the right show the GFAP^+^ cell number of the Dentate Gyrus, CA3, and CA1 in n-3 PUFA, NAÏVE, and OLIVE OIL groups. **(C)** Representative fluorescence images of confocal optical sections representing lipofuscin autofluorescent cells in the Dentate Gyrus of n-3 PUFA group (arrowhead). Scale bar 50 μm. The bottom histograms show quantitative analysis of the lipofuscin-loaded cells in Dentate Gyrus, CA3 and CA1. ^*^p < 0.05, ^**^p ≤ 0.01, or ^***^p ≤ 0.001; Student's *T* test.

To verify whether the increased cell density observed in n-3 PUFA group was linked to a decrease of apoptotic cell death, the apoptotic marker activated caspase-3 was measured in DG, CA1, and CA3. The number of caspase-3^+^ cells was very low in the three hippocampal subfields, without any difference among groups. By contrast, several caspase-3^+^ cellular debris were scattered throughout DG, CA1 and especially CA3 subfields in all groups, with a marked reduction was observed in n-3 PUFA group (Figure 4A).

#### Hippocampal microglia

Microglial cells are responsible for maintaining the extracellular environment of the brain, by regulating uptake and degradation of non-functional or apoptotic material. Reduced microglia self-renewal capacity and impaired clearance functions are reported in aging (Sierra et al., 2010; Gemma and Bachstetter, 2013). n-3 PUFA group displayed a significant increase in the number of microglial Iba1^+^ cells in comparison to both control groups in DG (n-3 PUFA vs. OLIVE OIL: *p* = 0.01; n-3 PUFA vs. NAÏVE: *p* = 0.0007), in CA3 (n-3 PUFA vs. OLIVE OIL: *p* < 0.0001; n-3 PUFA vs. NAÏVE: *p* = 0.0002), and in CA1 (n-3 PUFA vs. OLIVE OIL: *p* = 0.03; n-3 PUFA vs. NAÏVE: *p* = 0.007) (Figure 4A). More specifically, we observed in n-3 PUFA group an increase of the microglial cells phagocytosing apoptotic caspase-3^+^ neuronal debris in DG (n-3 PUFA vs. OLIVE OIL: *p* = 0.03; n-3 PUFA vs. NAÏVE: *p* = 0.04) and CA1 (n-3 PUFA vs. OLIVE OIL: *p* = 0.03; n-3 PUFA vs. NAÏVE: *p* = 0.01) when compared with the other experimental group (Supplementary Figure 1 and Figure 4A, square boxes). These results suggest that n-3 PUFA supplementation increases the phagocytosing microglial population, likely providing a protective mechanism during aging.

#### Hippocampal astroglia reactivity

A typical biomarker of the aging brain is the astrocyte hypertrophy with increased GFAP expression (Salminen et al., 2011). In n-3 PUFA group, GFAP immunoreactivity was significantly reduced in comparison to both control groups in DG (n-3 PUFA vs. OLIVE OIL: *p* = 0.0002; n-3 PUFA vs. NAÏVE: *p* = 0.0002) and CA3 (n-3 PUFA vs. OLIVE OIL: *p* = 0.002; n-3 PUFA vs. NAÏVE: *p* = 0.001), but not in CA1 (n-3 PUFA vs. OLIVE OIL: *p* = 0.43; n-3 PUFA vs. NAÏVE: *p* = 0.70) (Figure 4B). These results indicate that n-3 PUFA supplementation markedly reduced astrocytosis.

#### Hippocampal lipofuscin deposits

The non-specific lipofuscin deposits in neurons are a prominent sign of aging and their low levels are considered a measure of “cellular health” (Kempermann et al., 2002). In n-3 PUFA group the number of lipofuscin autofluorescent cells was significantly reduced in all hippocampal subfields in comparison to both control groups (DG: n-3 PUFA vs. OLIVE OIL: *p* = 0.04; n-3 PUFA vs. NAÏVE: *p* = 0.01; CA3: n-3 PUFA vs. OLIVE OIL: *p* = 0.009; n-3 PUFA vs. NAÏVE: *p* = 0.0009; CA1: n-3 PUFA vs. OLIVE OIL: *p* = 0.0006; n-3 PUFA vs. NAÏVE: *p* = 0.0007) (Figure 4C). These data indicate that n-3 PUFA supplementation resulted in ameliorated neuronal status promoting a decreased cellular degeneration and consequent neuronal loss.

#### Hippocampal neurogenesis

**Figure 5:**
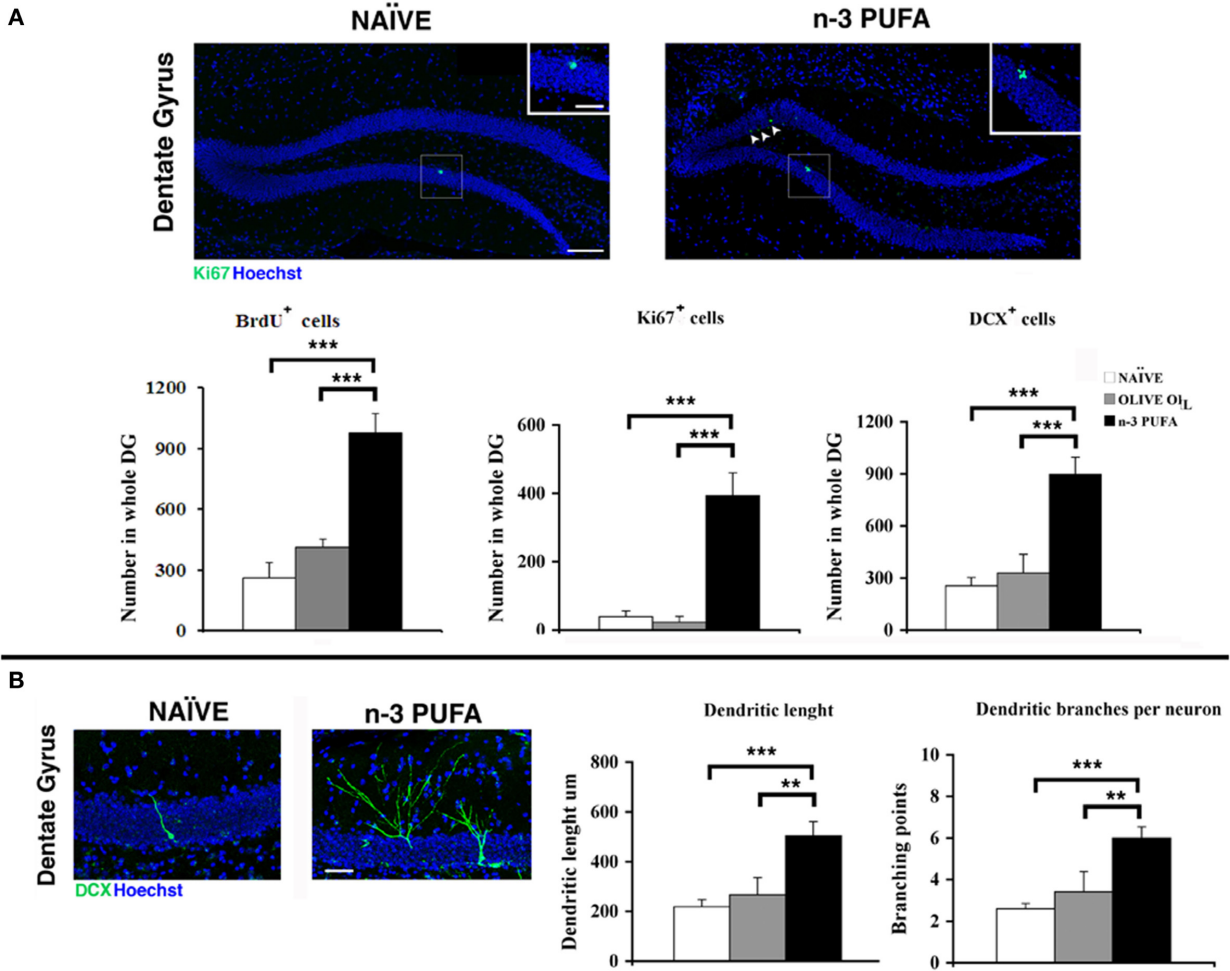
**n-3 PUFA effects on hippocampal neurogenesis. (A)** Representative images showing the increase of the Ki67^+^ cells in the dentate gyrus of the mice treated with n-3 PUFA, when compared with NAÏVE group. At higher magnification (square boxes) the presence of a cluster of Ki67^+^ cells, which is a sign of intense proliferative activity can be noted for the n-3 PUFA treated mice (right panel), but not for the NAÏVE mice (left panel). Scale bars 50 μm and 25 μm. The histograms show the increase of BrdU^±^, Ki67^+^ and DCX^+^ cells in the n-3 PUFA mice, when compared with NAÏVE and OLIVE OIL mice. **(B)** Representative morphology of DCX cells, analyzed from NAÏVE and n-3 PUFA experimental groups, showing an increase of dendritic complexity in the n-3 PUFA neuronal progenitors. Scale bar 50 μm. Quantification of dendritic length and dendritic branching points measured in NAÏVE, OLIVE OIL, and n-3 PUFA experimental groups. ^**^p ≤ 0.01, or ^***^p ≤ 0.001; Student's *T* test.

***Cell proliferation.*** The subgranular zone (SGZ) of the hippocampal DG constitutes one of the two neurogenic niches of the adult brain along with the subventricular zone (SVZ) (Eriksson et al., 1998). In SGZ, newborn cells proliferate from neural stem cells and give rise to immature neurons which migrate into the inner part of granule cell layer, where they mature and integrate into the pre-existing circuitry by receiving inputs from the entorhinal cortex, and extending projections into the CA3 (Zainuddin and Thuret, 2012). In order to evaluate the effect of n-PUFA administration on the hippocampal neurogenesis of aged mice we investigated the proliferation and differentiation of newborn neurons in the three experimental groups. In the n-3 PUFA group the number of BrdU^+^ cells was significantly increased in comparison to both control groups (n-3 PUFA vs. OLIVE OIL: *p* = 0.004; n-3 PUFA vs. NAÏVE: *p* < 0.0001), while no significant difference was evident between OLIVE OIL and NAÏVE groups (Figure 5A). Similarly, a significantly higher number of Ki67^+^ cells (Scholzen and Gerdes, 2000) was found in n-3 PUFA group compared to both control groups (n-3 PUFA vs. OLIVE OIL: *p* < 0.0001; n-3 PUFA vs. NAÏVE: *p* < 0.0001). The number of Ki67^+^ cells found in OLIVE OIL and NAÏVE mice was not significantly different (Figure 5A). These data demonstrate that in the DG the n-3 PUFA supplementation was effective in strongly increasing the cellular proliferation rate of newly-generated neurons that was conversely barely detectable in NAÏVE and OLIVE OIL mice.

***Cell differentiation.*** In n-3 PUFA group an increased number of DCX^+^ cells in the SGZ of DG was found in comparison to OLIVE OIL (*p* = 0.001) and NAÏVE (*p* < 0.0001) groups, while no statistical difference was found between OLIVE OIL and NAÏVE groups (*p* = 0.48) (Figure 5A). To investigate whether n-3 PUFA supplementation resulted in altered morphology of early-differentiated DCX^+^ neuroblasts, in term of dendritic length and branching points, two-dimensional projections of confocal z-series obtained from DCX^+^ cells were analyzed. In n-3 PUFA group a significant increase of dendritic length (n-3 PUFA vs. OLIVE OIL: *p* = 0.001; n-3 PUFA vs. NAÏVE: *p* < 0.0001) and branching points (n-3 PUFA vs. OLIVE OIL: *p* = 0.001; n-3 PUFA vs. NAÏVE: *p* < 0.0001) was observed (Figure 5B). These data indicate that the n-3 PUFA supplementation significantly stimulated neuroblast differentiation leading to major dendritic complexity.

### Metabolic correlates

#### Free amino acids and carnitines

**Figure 6:**
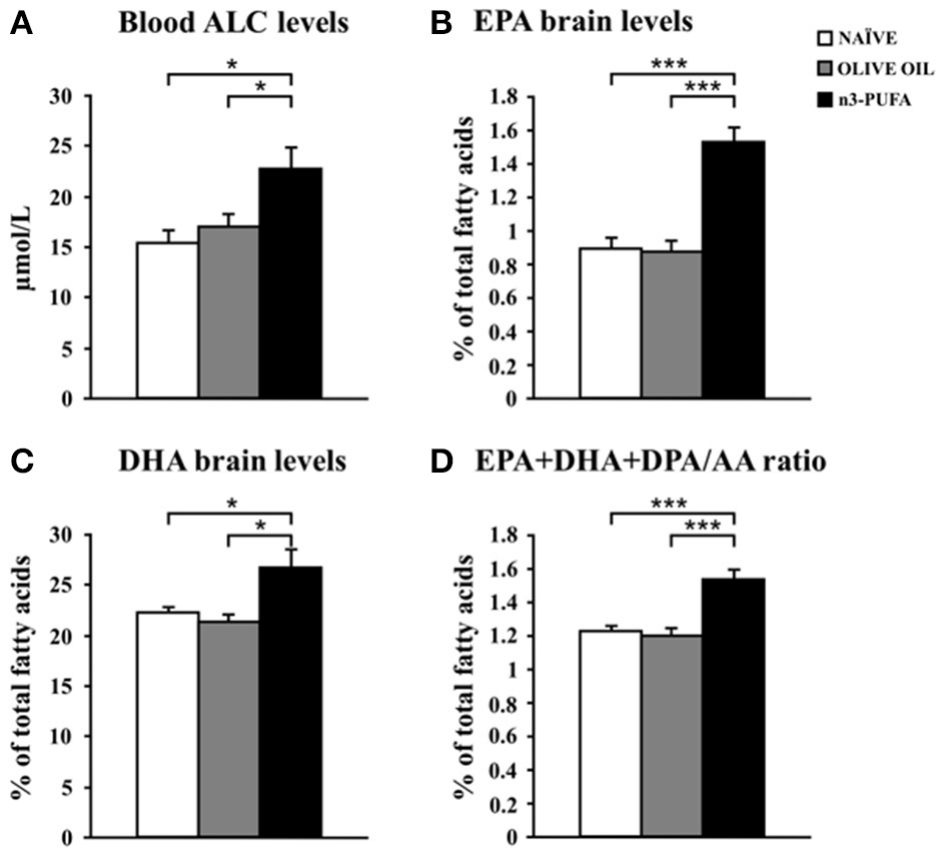
**n-3 PUFA effects on metabolic correlates. (A)** Average blood levels of acetylcarnitine (ALC) for the three experimental groups. **(B)** EPA brain levels. **(C)** DHA brain levels. **(D)** EPA+DHA+DPA/AA ratio. ^*^p < 0.05, or ^***^p < 0.001.

Blood levels of amino acids and carnitines are reported in Table 2. Supervised and Unsupervised Principal Component Analysis did not reveal any significant difference among the experimental groups. However, One-Way ANOVA on the acetylcarnitine (ALC) levels revealed a significant difference [*F*_(2, 12)_ = 5.52, *p* = 0.02] among groups. *Post-hoc* comparisons indicated that ALC blood levels were significantly higher in n-3 PUFA group in comparison to OLIVE OIL (*p* = 0.03) and NAÏVE (*p* = 0.02) (Figure 6A).

**Table 2:** **Blood concentrations of amino acids and carnitines for each experimental group are indicated**.

**Amino acids concentrations**	**NAÏVE**	**OLIVE OIL**	**n-3 PUFA**
Proline	86.81 ± 6.42	81.85 ± 6.41	91.75 ± 13.28
Valine	79.68 ± 5.86	100.56 ± 2.13	86.24 ± 13.23
Leucine/Isoleucine/Hydroxyproline	132.01 ± 9.76	130.61 ± 14.02	137.12 ± 13.19
Ornithine	53.24 ± 12.18	44.29 ± 4.62	54.22 ± 13.17
Methionine	26.14 ± 6.23	24.86 ± 1.87	24.55 ± 2.19
Phenylalanine	40.94 ± 3.29	44.73 ± 2.04	42.79 ± 3.69
Arginine	90.05 ± 4.46	77.80 ± 0.85	89.65 ± 5.82
Citrulline	36.87 ± 2.76	34.86 ± 4.87	39.07 ± 7.00
Tyrosine	44.46 ± 7.96	41.25 ± 2.80	40.84 ± 3.59
Glycine	632.28 ± 128.26	609.72 ± 112.52	619.95 ± 67.96
Alanine	57.92 ± 9.73	69.77 ± 2.73	74.46 ± 8.89
Serine	7.35 ± 0.74	9.48 ± 1.36	8.29 ± 2.62
Threonine	27.41 ± 4.98	26.54 ± 2.39	28.38 ± 4.20
Asparagine	4.10 ± 0.91	3.42 ± 0.32	3.27 ± 0.60
Aspartate	4.99 ± 0.36	4.75 ± 0.61	6.40 ± 0.51
Lysine/Glicine	1592.42 ± 285.89	1583.62 ± 200.26	1710.13 ± 136.14
Glutamate	34.97 ± 3.25	35.51 ± 1.57	31.40 ± 1.59
Histidine	52.12 ± 3.69	53.23 ± 1.10	54.65 ± 4.99
t**Carnitines concentrations**			
Carnitine free	14.62 ± 2.05	17.97 ± 1.11	15.31 ± 1.98
Acetylcarnitine^*^	15.32 ± 1.38	17.02 ± 1.24	22.76 ± 2.19
Propionylcarnitine	0.86 ± 0.08	1.05 ± 0.07	0.92 ± 0.08
Butyrylcarnitine	0.15 ± 0.01	0.11 ± 0.03	0.12 ± 0.03
Iovalerylcarnitine	0.06 ± 0.01	0.07 ± 0.01	0.09 ± 0.01
3-Hydroxybutyrylcarnitine/ Malonylcarnitine	0.13 ± 0.02	0.15 ± 0.01	0.23 ± 0.07
3-Hydroxyisovalerylcarnitine/ Methylmalonylcarnitine	0.13 ± 0.03	0.15 ± 0.01	0.16 ± 0.01
Glutarylcarnitine/3-Hydroxyhexanoylcarnitine	0.22 ± 0.02	0.2 ± 0.03	0.23 ± 0.02
Adipylcarnitine	0.09 ± 0.02	0.09 ± 0.01	0.11 ± 0.01
Tetradecenoylcarnitine	0.06 ± 0.01	0.07 ± 0.01	0.09 ± 0.03
Myristoylcarnitine	0.17 ± 0.03	0.14 ± 0.01	0.17 ± 0.03
Hexadecenoylcarnitine	0.13 ± 0.02	0.12 ± 0.01	0.15 ± 0.04
Palmitoylcarnitine	1.09 ± 0.07	1.08 ± 0.04	1.19 ± 0.07
Linoleylcarnitine	0.08 ± 0.01	0.08 ± 0.01	0.08 ± 0.01
Oleylcarnitine	0.42 ± 0.02	0.49 ± 0.02	0.50 ± 0.08
Stearoylcarnitine	0.21 ± 0.01	0.22 ± 0.01	0.24 ± 0.01

Values represent mean ± s.e.m.

* p < 0.05.

No significant differences were found for amino acids blood levels.

#### N-3 PUFA brain levels

EPA, DHA, and DPA were measured as the major n-3 PUFA components of brain cell membranes. Moreover, the EPA+DHA+DPA/Arachidonic Acid (AA) ratio was assessed due to its role in cognitive dysfunction and neuroinflammation (Rao et al., 2007; Labrousse et al., 2012). EPA and DHA, but not DPA, levels increased in n-3 PUFA group in comparison to NAÏVE and OLIVE OIL groups, as revealed by one-way ANOVAs on EPA and DHA levels [EPA: *F*_(2, 12)_ = 26.77, *p* < 0.0001; DHA: *F*_(2, 12)_ = 6.21, *p* = 0.01; DPA: *F*_(2, 12)_ = 1.85, *p* = 0.20]. *Post-hoc* comparisons indicated that n-3 PUFA mice exhibited higher EPA (n-3 PUFA vs. NAÏVE *p* = 0.0001; n-3 PUFA vs. OLIVE OIL *p* < 0.0001) and DHA (n-3 PUFA vs. NAÏVE *p* = 0.04; n-3 PUFA vs. OLIVE OIL *p* = 0.02) levels (Figures 6B,C). No significant differences were found by comparing data of NAÏVE and OLIVE OIL mice. Moreover, One-Way ANOVA on the EPA+DHA+DPA/AA ratio revealed significant differences among groups [*F*_(2, 12)_ = 17.54, *p* = 0.0003]. *Post-hoc* comparisons indicated a significant increase of EPA+DHA+DPA/AA ratio in n-3 PUFA group in comparison to NAÏVE (*p* = 0.0005) and OLIVE OIL (*p* = 0.001) groups (Figure 6D).

## Discussion

The present data consistently demonstrate the beneficial effects of n-3 PUFA supplementation on hippocampal resilience to aging. In particular, our study indicates that a mixture of n-3 PUFA containing EPA, DHA, and DPA may improve hippocampal functioning in C57B/6 aged mice. Interestingly, n-3 PUFA group exhibited improved performances in all tasks tapping hippocampal functions, as recognition memory (ORT), spatial performances (SYM, MWM), and aversive responses (CFC), without differences in anxiety levels (EPM) in comparison to OLIVE OIL and NAÏVE groups. The marked improvement of hippocampal cognitive functions occurred in the context of an enhanced cellular plasticity. In fact, n-3 PUFA noticeably increased neurogenesis, dendritic arborization of DG newborn neurons, hippocampal volumes and cell density. These findings, well corroborated by the decrease in apoptosis, astrocytosis and lipofuscin deposits as well as the increase in microglial cells observed in n-3 PUFA group, demonstrate the specific neuroprotective effects of n-3 PUFA on hippocampal circuits. In accordance with the statement by Kempermann et al. (2002), n-3 PUFA group's hippocampus has thus to be considered as *healthier* for a general decrease in neurodegeneration (lipofuscin deposits, astroglia reactivity, caspase-3^+^ cells) and an increase in regeneration (neurogenesis) and clearance (microglial cells). Importantly, also the increased levels of metabolic correlates (blood ALC, cerebral n-3 PUFA) of n-3 PUFA treated mice point toward an effective neuroprotective action of these fatty acids. Furthermore, we demonstrated that the signs of neuronal aging can be diminished by enhancing n-3 PUFA intake, even when the supplementation starts at late age. Thus, this category of fatty acids exerts not only an acute but also a sustained effect on brain plasticity.

An interesting distinctive feature of the present study is the use of commonly available supplements, as the easy-to-buy n-3 PUFA mixture and frequently used olive oil. This accounts for the ecological validity of the present results increasing their possibility of generalization.

A further strength point of this research is the analysis in the same aged animals of hippocampal mnesic functions, neurogenic and glial responses, volumetric changes, and metabolic correlates. Our findings expand those of the few animal studies addressing n-3 PUFA effects on cognitive functions during aging. Namely, it has been reported that EPA, DPA and/or DHA supplementation delays cognitive decline in n-3 PUFA deficient aged animals (Gamoh et al., 2001; Carrié et al., 2002), in senescence-accelerated prone 8 mice (Petursdottir et al., 2008) and in aged rodents (Jiang et al., 2009; Kelly et al., 2011; Labrousse et al., 2012). The present behavioral data nicely fit also with the few studies demonstrating the n-3 PUFA efficacy on slowing cognitive decline in older people (Danthiir et al., 2011). However, this is the first research associating behavioral, neurochemical and neurogenic aspects in the general framework of the hippocampal functionality in the presence of n-3 PUFA supplementation during aging. Moreover, in the present study we report for the first time n-3 PUFA enhancing effects on dendritic length and branching complexity of newborn DG neurons. Interestingly, along with substantial improvements in behavioral performance, in n-3 PUFA group the proliferation rate of newly-generated DG cells was potentiated with a number of newborn neurons markedly higher than the control groups. Furthermore, the n-3 PUFA supplementation stimulated neuroblasts differentiation leading to major dendritic length and branching complexity of newborn DG neurons. These observations fully fit with reversal of decreased neurogenesis induced by n-3 PUFA treatment even at old age (Dyall et al., 2007, 2010) and also with the neurite enhancement (neuritogenesis) described in dorsal root ganglia of EPA or DHA supplemented aged rats (Robson et al., 2010). The mechanisms of the n-3 PUFA neurogenic action have still not been conclusively described. n-3 PUFA supplementation might increase the neuronal differentiation and neuritogenesis through a reduction of inflammation. Such an anti-inflammatory activity could be exerted by a metabolization of n-3 PUFA to neuroprotective mediators, such as neuroprotectin D1. Furthermore, n-3 PUFA might increase the signaling factors involved in neurogenesis, such as BDNF, CREB, or CaMKII (Maruszak et al., 2014), and the expression of genes involved in neurogenesis and neuritogenesis regulated at the transcriptional level (Dyall et al., 2010). Finally, n-3 PUFA might exert their bioactivity even through syntaxin 3 that mediates membrane expansion at the growth cone given rise to neurite outgrowth (He et al., 2009).

An important aspect of the increased n-3 PUFA-induced neurogenesis was its occurrence on the background of signs of decreased neurodegeneration, as indicated by the increased microglial phagocytosis and decreased astrocytosis and lipofuscin deposits. In fact, the recent focus on the interdependent processes linking microglia and neurogenesis evidenced, not only the microglia role in phagocytosing apoptotic cells, but also their ability in secreting factors that influence proliferation, differentiation, and survival of newborn cells, either by a direct instructive role or through secretion of neurotrophic factors (Gemma and Bachstetter, 2013). Interestingly, n-3 PUFA, and mainly DHA, increase the level of BDNF, which is predominantly synthesized by hippocampal neurons (Jiang et al., 2009). BDNF can activate synaptic proteins, such as synapsin-1 that increases the synthesis of synaptic membranes, and leads to elevated levels of phosphatides and specific pre- and post-synaptic proteins. Via this pathway n-3 PUFA increase the number of dendritic spines and possibly synapses on hippocampal neurons, particularly the excitatory glutamatergic synapses involved in learning and memory (Janssen and Kiliaan, 2014).

Despite the vast literature on n-3 PUFA effects on brain structure and function, lipofuscin load has never been studied in n-3 PUFA supplemented subjects. The reduced lipofuscin deposits exhibited by n-3 PUFA group in the whole hippocampus indicated a decreased age-dependent degeneration, likely concurring to the sustained (even at old age) induction of neurogenesis by n-3 PUFA dietary enrichment. Analogously, caloric restriction, with its robust effects on longevity, potentiates hippocampal neurogenesis and reduces lipofuscin load (Moore et al., 1995; Szweda et al., 2003).

The diffuse astrocytosis occurring with age was markedly reduced in n-3 PUFA group, indicating that an efficient astroglial function should be maintained to prevent brain aging. Given the high DHA concentrations in their membranes, astrocytes are optimal targets of n-3 PUFA action in the brain. Interestingly, n-3 PUFA deficiency worsens age-induced hippocampal astrocytosis and promotes neuroinflammation (Layé, 2010; Latour et al., 2013). On such a basis the reduction of astroglia reactivity by n-3 PUFA supplementation demonstrated here could lead to a reduction of pro-inflammatory cytokines.

Even if the restored neurogenesis could be related to the increased hippocampal volumes of n-3 PUFA group, such a factor, as far as markedly significant, does not appear to be enough to explain the whole hippocampal volumetric increase. In fact, the neurogenic proliferation is a specific prerogative of dentate gyrus, while the volumetric enlargement was found in all hippocampal subfields. The enhanced hippocampal volume may rather be related to a prevented neuronal loss linked to the n-3 PUFA neuroprotective properties. This hypothesis takes into account the reduction in apoptosis, glial degeneration and oxidative stress, and the parallel increase in neuronal and microglial density observed in the n-3 PUFA group. Many studies highlighted n-3 PUFA efficacy in preventing hippocampal neuronal loss in AD-like neurodegenerative models (Su, 2010; Hooijmans et al., 2012; Denis et al., 2013; Luchtman and Song, 2013). In addition, the few human studies addressing the relations between n-3 PUFA intake and brain volumes converge on detrimental effects of n-3 PUFA deficiency and beneficial effects of their presence. Namely, in recurrent affective disorders, including unipolar and bipolar depression, the n-3 PUFA deficiency is associated with reduction of the gray matter volume in the prefrontal cortex inducing in turn alterations in cortico-limbic projections (McNamara, 2010). Conversely, a positive association between n-3 PUFA intake and gray matter volumes in hippocampus, amygdala and anterior cingulate cortex were found in healthy subjects (Conklin et al., 2007). More recently, EPA and DHA intake resulted positively correlated with gray and white matter volumes and cognitive performances in elderly subjects (Titova et al., 2013; Virtanen et al., 2013).

Ultimately, how may the morphological changes here observed be linked to the significant behavioral improvements induced by n-3 PUFA supplementation? The increase of hippocampal neuronal density induced by n-3 PUFA may be at least in part related to the increase of phagocytic microglial cells and to the concomitant reduction of astrogliosis, which in turn may favor the cellular homeostasis and the hippocampal functionality within the aged brain, thus finally improving mnesic abilities. Indeed, it has been shown that aging is associated with senescence, reduced self-renewal and impaired clearance functions of microglia, resulting in a progressive impairment of the brain cellular processes (Streit, 2006; Sierra et al., 2007).

**Figure 7:**
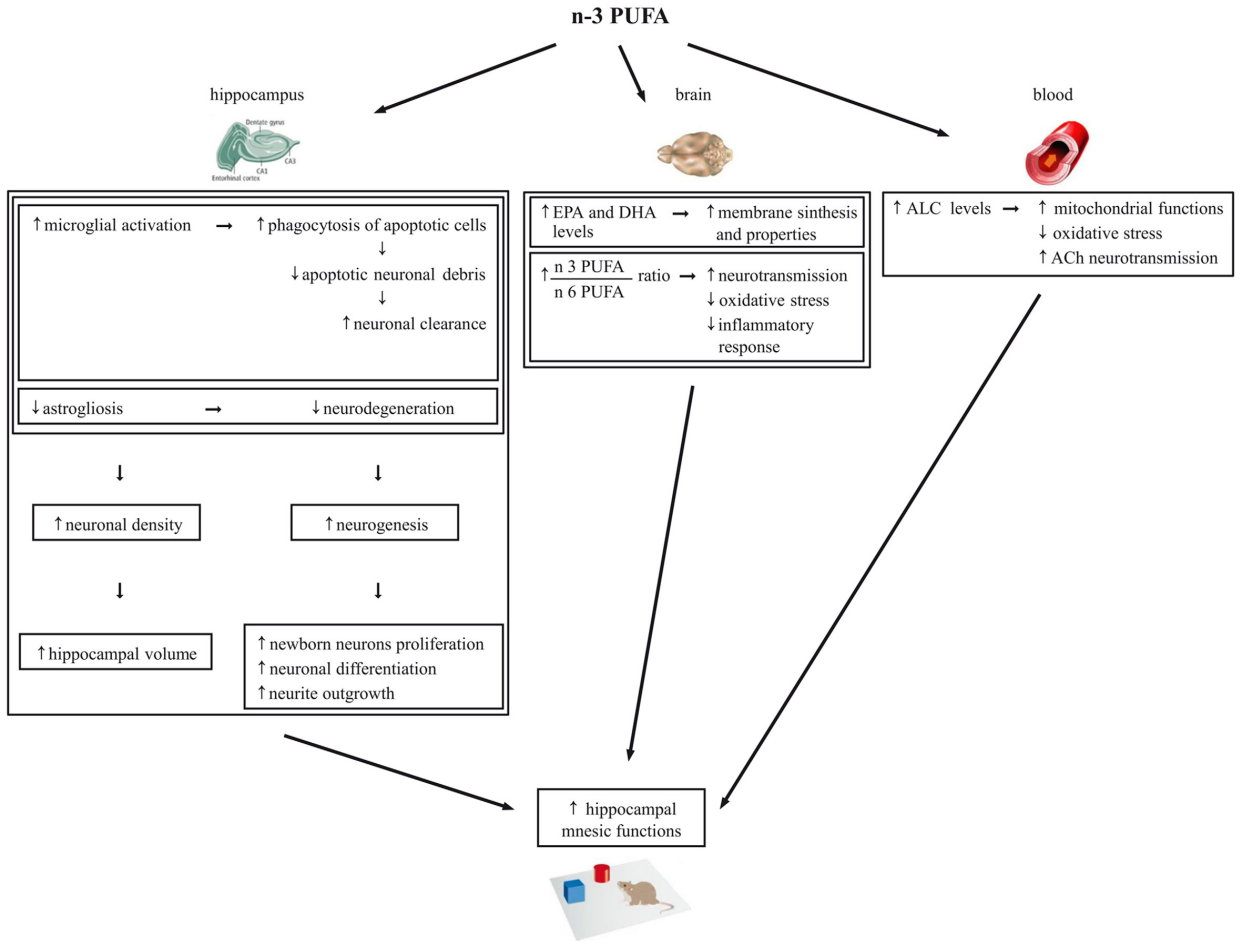
**Schematic representation of the cellular mechanisms underlying the beneficial effects of n-3 PUFA supplementation on hippocampal-dependent mnesic functions in aged mice**.

Neurogenesis is required for some types of learning and memory, and its rate decreases during aging in humans, non-human primates, and rodents (Maruszak et al., 2014). Microglia are essential component of the neurogenic niche in the DG of the adult hippocampus (Gemma and Bachstetter, 2013) and have an important function in phagocytosis of apoptotic cells during the first days of their life. Thus, they provide an essential pruning allowing a more efficient differentiation and integration of newborn neurons into the existing circuits (Sierra et al., 2010). On such a basis, we hypothesize that the increase of microglia may concur to the increment of neurogenesis observed in the n-3 PUFA group (Figure 7).

Also with regards to metabolic findings we observed increased EPA and DHA brain levels and EPA+DHA+DPA/AA ratio in n-3 PUFA group, in line with Gamoh et al. (2001) and Labrousse et al. (2012). As known, EPA, DPA and especially DHA are key blocks for brain cell membrane synthesis and properties, including fluidity, flexibility, permeability and neurotransmission regulation (Crupi et al., 2013). Moreover, in the brain DHA and AA are continuously released from membrane phospholipids and create a constant turn-over of PUFA between brain and plasma to be daily balanced by the diet (Luchtman and Song, 2013). The n-3:n-6 PUFA ratio is important for essential neurobiological functions like neurotransmission, as well as for the equilibrium of the bioactive metabolites produced from n-3 PUFA that are involved in the oxidative stress and inflammatory response (Denis et al., 2013). Interestingly, the relative lack of n-3 PUFA featuring the Western diet results in a chronic n-3 PUFA deficit in brain membranes and overproduction of AA derivatives that favor the emergence of the a pro-inflammatory status characteristic of brain aging (Denis et al., 2013). On such a basis, we hypothesize that the increase of EPA+DHA+DPA/AA ratio observed in the brain of n-3 PUFA group results in a reduction of the oxidative stress and inflammatory responses and in an enhancement of neurotransmission that in turn contribute in retaining better mnesic performances (Figure 7).

Finally, it has to be taken into account that the ameliorated mnesic performances of n-3 PUFA group were associated to high ALC blood levels, without differences in the other carnitines and amino acids analyzed. Crossing the blood-brain barrier, ALC shuttles acetyl groups and fatty acids into brain cell mitochondria for energy production and acts as direct anti-oxidant (Kobayashi et al., 2010; Goo et al., 2012). Remarkably, ALC improves cognitive deficits in aged or demented human subjects (Ames and Liu, 2004; Mancuso et al., 2007; Glade, 2010; Malaguarnera, 2012). Preclinical studies indicate that ALC counteracts cognitive decline even in the presence of β-amyloid toxicity (Kaur et al., 2001; Dhitavat et al., 2005; Abdul et al., 2006; Barhwal et al., 2009; Suchy et al., 2009). Moreover, ALC improves mnesic capacities, restores serum, heart, muscle and brain carnitine levels, and improves the cholinergic neurotransmission dysregulated by aging (Kobayashi et al., 2010). Notably, in animal studies also n-3 PUFA dietary supplementation improves cholinergic transmission in the brain (Willis et al., 2009). Moreover, additive effects of ALC and PUFA supplementation in reducing age-related retinal degeneration (Feher et al., 2005) and brain damages caused by oxidative stress (Liu et al., 2002) have been reported. Thus, our findings indicating enhanced ALC levels following n-3 PUFA supplementation not only support the ALC neuroprotective efficacy, but for the first time demonstrate that an enhanced n-3 PUFA intake is able to increase ALC levels. This intriguing association may lead to the described cognitive improvement by improving mitochondrial functions and reducing oxidative stress but likely even by enhancing cholinergic transmission (Figure 7).

In conclusion, our study suggests that n-3 PUFA are able to get an aged hippocampus to be “younger” and more functional by multiple mechanisms, as summarized in Figure 7. Consequently, n-3 PUFA appear ideal candidates for cognition-enhancing nutritional interventions aimed to promote successful and healthy aging. This issue is of growing relevance, given the pressing question of how the elderly can maintain their cognitive functions in the Western population whose life expectancy increasingly raises.

Future studies investigating the cellular and molecular mechanisms through which PUFA supplementation acts will be useful for understanding its functional significance in counteracting brain aging.

## Author contributions

Debora Cutuli, Gianfranco Spalletta, Carlo Caltagirone, Stefano Farioli-Vecchioli, and Laura Petrosini designed research; Debora Cutuli, Paola De Bartolo, Paola Caporali, Daniela Laricchiuta, Francesca Foti, Maurizio Ronci, Claudia Rossi, Cristina Neri, Stefano Farioli-Vecchioli, and Laura Petrosini performed research; Debora Cutuli, Paola De Bartolo, Paola Caporali, Maurizio Ronci, Cristina Neri, Stefano Farioli-Vecchioli and Laura Petrosini analyzed data; Debora Cutuli, Maurizio Ronci, Cristina Neri, Stefano Farioli-Vecchioli, and Laura Petrosini wrote the paper.

### Conflict of interest statement

The authors declare that the research was conducted in the absence of any commercial or financial relationships that could be construed as a potential conflict of interest.

## Supplementary material

Supplementary Figure 1 **The histograms show the phagocytosing Iba1^+^ cell number of the Dentate Gyrus, CA3, and CA1 in n-3 PUFA, NAÏVE, and OLIVE OIL groups**.
