# Supplementary figures and images for: n-3 polyunsaturated fatty acids supplementation enhances hippocampal functionality in aged mice
Source: Front Aging Neurosci. 2014 Aug 25;6:220. doi: 10.3389/fnagi.2014.00220 (PMC4142709; doi:10.3389/fnagi.2014.00220)

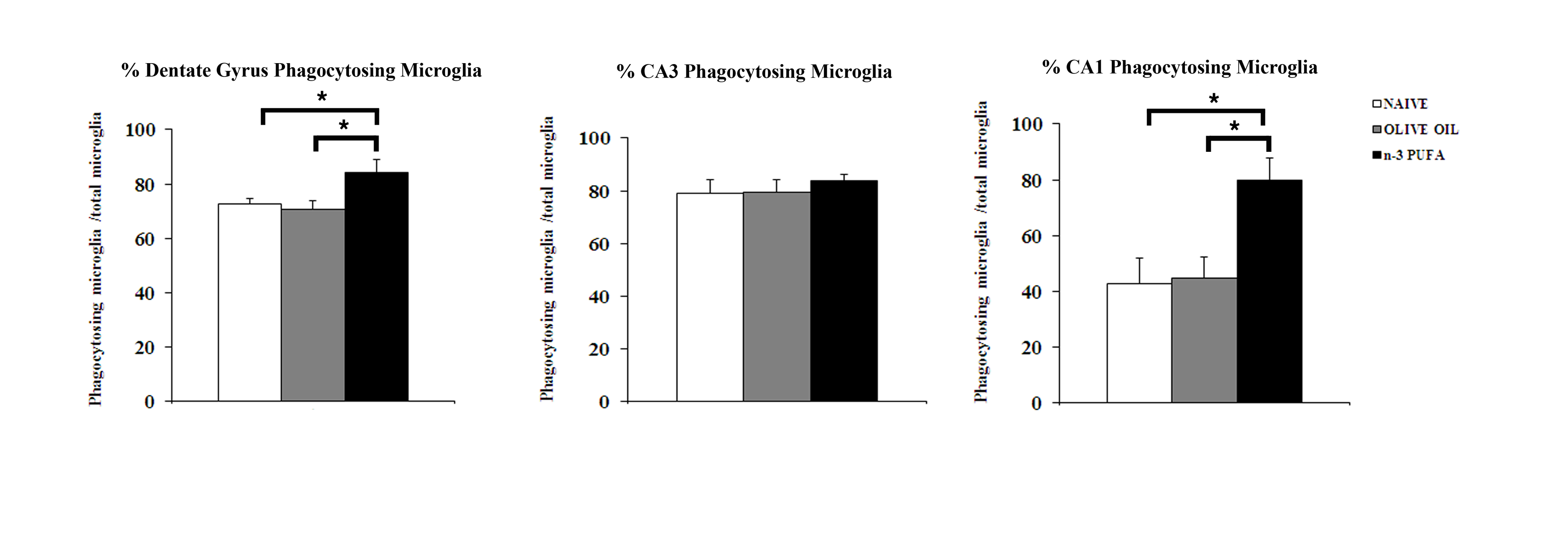

Supplement: Supplementary Figure 1 — The histograms show the phagocytosing Iba1+ cell number of the Dentate Gyrus, CA3, and CA1 in n-3 PUFA, NAÏVE, and OLIVE OIL groups. [file Image1.TIF]
